# Formation of Neutrophil Extracellular Traps under Low Oxygen Level

**DOI:** 10.3389/fimmu.2016.00518

**Published:** 2016-11-25

**Authors:** Katja Branitzki-Heinemann, Helene Möllerherm, Lena Völlger, Diab M. Husein, Nicole de Buhr, Stefanie Blodkamp, Friederike Reuner, Graham Brogden, Hassan Y. Naim, Maren von Köckritz-Blickwede

**Affiliations:** ^1^Department of Physiological Chemistry, University for Veterinary Medicine Hannover, Hanover, Germany; ^2^Research Center for Emerging Infections and Zoonoses (RIZ), University for Veterinary Medicine Hannover, Hanover, Germany

**Keywords:** neutrophils, innate immunity, neutrophil extracellular traps, hypoxia, HIF-1α, cholesterol

## Abstract

Since their discovery, neutrophil extracellular traps (NETs) have been characterized as a fundamental host innate immune defense mechanism. Conversely, excessive NET-release may have a variety of detrimental consequences for the host. A fine balance between NET formation and elimination is necessary to sustain a protective effect during an infectious challenge. Our own recently published data revealed that stabilization of hypoxia-inducible factor 1α (HIF-1α) by the iron chelating HIF-1α-agonist desferoxamine or AKB-4924 enhanced the release of phagocyte extracellular traps. Since HIF-1α is a global regulator of the cellular response to low oxygen, we hypothesized that NET formation may be similarly increased under low oxygen conditions. Hypoxia occurs in tissues during infection or inflammation, mostly due to overconsumption of oxygen by pathogens and recruited immune cells. Therefore, experiments were performed to characterize the formation of NETs under hypoxic oxygen conditions compared to normoxia. Human blood-derived neutrophils were isolated and incubated under normoxic (21%) oxygen level and compared to hypoxic (1%) conditions. Dissolved oxygen levels were monitored in the primary cell culture using a Fibox4-PSt3 measurement system. The formation of NETs was quantified by fluorescence microscopy in response to the known NET-inducer phorbol 12-myristate 13-acetate (PMA) or *Staphylococcus (S.) aureus* wild-type and a nuclease-deficient mutant. In contrast to our hypothesis, spontaneous NET formation of neutrophils incubated under hypoxia was distinctly reduced compared to control neutrophils incubated under normoxia. Furthermore, neutrophils incubated under hypoxia showed significantly reduced formation of NETs in response to PMA. Gene expression analysis revealed that mRNA level of *hif-1*α as well as *hif-1*α target genes was not altered. However, in good correlation to the decreased NET formation under hypoxia, the cholesterol content of the neutrophils was significantly increased under hypoxia. Interestingly, NET formation in response to viable *S. aureus* wild-type or nuclease-deficient strain was retained under hypoxia. Our results lead to the conclusion that hypoxia is not the ideal tool to analyze HIF-1α in neutrophils. However, the data clearly suggest that neutrophils react differently under hypoxia compared to normoxia and thereby highlight the importance of the usage of physiological relevant oxygen level when studying neutrophil functions.

## Introduction

Neutrophils belong to the first line of defense of the innate immune system against various pathogens including bacteria, fungi, and protozoa. Besides degranulation and the intracellular killing of pathogens, neutrophils are able to entrap and kill pathogens by the release of extracellular structures, so-called neutrophil extracellular traps (NETs) (1). NETs are formed upon activation in response to a wide range of stimuli, like interferon-α, interleukin-8 (IL-8), the pharmacological agent phorbol 12-myristate 13-acetate (PMA), as well as numerous microbes and their products [reviewed by von Köckritz-Blickwede and Nizet (2)]. The formation of NETs is characterized by the disruption of the nuclear membrane, chromatin decondensation, and the mixing of nuclear contents with cytoplasmic and granular proteins. As a final step, the nuclear and granular components are released into the extracellular space (3). The fibrous DNA functions as a backbone in which histones, proteases [e.g., myeloperoxidase (MPO) and elastase], and antimicrobial peptides (AMPs) (e.g., cathelicidins) reside mediating their antimicrobial activity (4). The transcriptional regulation and intracellular signaling pathways of NET generation has not yet been fully investigated.

Generation of reactive oxygen species (ROS) is a key event for NET formation, and NET-based antimicrobial activity crucially depends on the formation of ROS through the membrane-bound NADPH oxidase enzyme complex as well as MPO (3, 5–9). Consequently, the most frequently used pathway to induce NETs is triggered by PMA, a protein kinase C (PKC) activator (1, 10, 11). That, in turn, activates the NADPH oxidase complex-subunit p47^Phox^ (12) and thus strongly supports its activation. The produced superoxide anions serve as a starting product for additional ROS (13). Blocking the NADPH oxidase inhibits ROS generation and NET-release, respectively (14).

The downstream effects of ROS are extremely broad and range from the induction of NF-κB signaling (15), to peroxidation of phospholipids (16), or activation of the cell death receptor (17). Another interesting target of ROS is the hypoxia-inducible factor 1α (HIF-1α) (18).

HIF-1α was initially known to act as a transcriptional activator functioning as a master regulator of cellular and systemic oxygen homeostasis. Nowadays, HIF-1α was additionally shown to play a role in the production of defense factors and to improve the bactericidal activity of myeloid cells (19, 20). Peyssonnaux and colleagues demonstrated in 2005 for the first time that HIF-1α expression regulates the antibacterial capacity of phagocytes focusing on neutrophils and macrophages. HIF-1α was induced by different bacterial pathogens including *Staphylococcus (S.) aureus* and *Streptococcus pyogenes*, even under normal oxygen levels (normoxia), and regulated the production of key immune effector molecules. Although the full spectrum of HIF-1α downstream targets remains to be determined, the expression of a number of molecular effectors of host defense, including AMPs, TNF-α, and the granule proteases cathepsin G and elastase, significantly correlated with HIF-1α levels (19). Mice lacking HIF-1α in their myeloid cell lineage showed decreased bactericidal activity and were not able to restrict a systemic spread of an infection from its initial tissue (19).

It was already shown that HIF-1α is crucial in the formation of extracellular traps in mast cells [mast cell extracellular traps (MCETs)]. Augmentation of HIF-1α-activity resulted in a boosting of the antimicrobial activity of human and murine mast cells by inducing extracellular trap formation (21). At the same time, HIF-1α-deficient mast cells exhibited reduced antimicrobial activity and ability to form extracellular traps. Recently, it was reported that the mammalian target of rapamycin (mTOR), a highly conserved PI3K-like serine/threonine kinase and a posttranscriptional regulator of HIF-1α protein expression, regulates the formation of NETs (22). As mTOR kinase is known as a key regulator of autophagy in many mammalian cells including neutrophils, it is hypothesized that mTOR plays a regulatory role in NET-release by regulating autophagic activity (23). Interestingly, McInturff et al. (22) also demonstrated that the iron chelating HIF-1α agonist cobalt chloride (CoCl_2_) triggered NET formation (22). Several authors discussed that the regulation of ROS generation could be a key factor in these HIF-1α- and mTOR-mediated processes (22–24). Our own data confirm the hypothesis that HIF-1α might be involved in the formation of NETs: the HIF-1α-agonist desferoxamine enhanced the release of extracellular traps in human and bovine neutrophils in a ROS-dependent manner (25).

Although the importance of HIF-1α in the formation of NETs has already been stated, the impact of hypoxia on NET generation still needs to be clarified. Hypoxia was found to be able to enhance bactericidal activities of human neutrophils, increase their chemotactic, phagocytic, and respiratory burst capacities, and protect them from apoptosis (19, 26–29). Based on the described literature it may be hypothesized that NET formation increases under low oxygen conditions similarly to that shown by HIF-1α stabilizing agents.

## Materials and Methods

### Bacterial Strains

*Staphylococcus aureus* strain USA 300 wildtype (wt) (LAC AH 1263) and its nuclease mutant (Δnuc) (LAC AH 1680) (30) were used in this study. *S. aureus* was grown in brain heart infusion (BHI) medium at 37°C with shaking. Fresh overnight cultures were diluted 1:100 in BHI and then grown to mid-exponential growth phase (OD_600_ = 0.7) until usage. Heat inactivation was performed for 30 min in 95°C hot water.

### Neutrophil Isolation

Human blood-derived neutrophils were isolated from healthy donors in agreement with the local ethical board by density gradient centrifugation at 500 × *g* using PolymorphPrep (Axis-Shield, Oslo, Norway) as previously described (31). Then, neutrophils were resuspended in RPMI 1640 (PAA, Freiburg, Germany) and plated on poly-l-lysine (# P4707, Sigma-Aldrich) coated coverslips at a concentration of 2 × 10^5^ cells/well in 48-well plates or 5 × 10^5^ cells/well in 24-well plates (Nunc, Germany).

### Oxygen Measurements

Oxygen measurements were performed as previously described (32) using a Fibox4-PSt3 measurement system in 24-well plates (Nunc, Germany). Importantly, oxygen was measured non-invasively and was not consumed during the process of measurement. Freshly isolated neutrophils from human blood were adapted to hypoxia. Using optical sensors (placed on the bottom of the wells in the medium), the dissolved oxygen level in the cell culture media was measured based on the oxygen-dependent quenching of phosphorescent probes (32–34). Oxygen measurements were performed over a time period of 5 h while the cells were incubated under hypoxic (7 mmHg, 1% O_2_) or normoxic (159 mmHg, 21% O_2_) conditions, respectively.

### NET Induction and Visualization

Neutrophils were preincubated under normoxic or hypoxic conditions for 2 h before they were subsequently infected with living or heat-inactivated bacteria (MOI 2) and incubated at 37°C and 5% CO_2_ for 3 h under the respective oxygen condition. As a positive control to stimulate NET formation, 25 nM PMA (Sigma, Hamburg, Germany) was used, while untreated neutrophils served as a negative control. Neutrophils were treated with 10 μg/ml diphenyleneiodonium (DPI) to block NADPH oxidase activity. Finally, cells were fixed with 4% paraformaldehyde (PFA; Roth, Germany).

Neutrophil extracellular traps were stained with an antibody against the histone–DNA complex (Millipore, mouse monoclonal anti DNA/Histone1 MAB 3864). Briefly, after blocking and permeabilization [2% BSA PBS + 0.2% Triton X-100 for 45 min at room temperature (RT)], samples were incubated for 1 h at RT with the primary antibody (2.2 mg/ml, diluted 1:5000 in PBS containing 2% BSA, 0.2% Triton X-100). An Alexa Fluor 488-conjugated goat anti-mouse antibody (Thermo Scientific; diluted 1:1000 in PBS containing 2% BSA, 0.2% Triton X-100) was used as a secondary antibody. Slides were embedded in ProlongGold^®^ antifade with DAPI (P36931, Molecular Probes) and analyzed by confocal fluorescence microscopy using a Leica TCS SP5 confocal microscope with a HCX PL APO 40× 0.75–1.25 oil immersion objective. Settings were adjusted in accordance to control preparations using the respective isotype control antibody. The total number of neutrophils and the number of neutrophils releasing NETs per field of view were counted in six representative images per sample.

### Lipid Isolation and Analysis

A total of 5 × 10^6^ neutrophils were incubated in a 1.5 ml reaction tube for 3 h at either 1 or 21% oxygen. Samples were washed twice with PBS, resuspended in chloroform–methanol (1:1), and lysed by passing cells through a 45 mm cannula syringe 15 times. Subsequent lipid isolation was performed as previously described (35).

Cholesterol content was analyzed with a Hitachi Chromaster HPLC using a Chromolith^®^ HighResolution RP-18 endcapped 100–4.6 mm column coupled to a 5–4.6 mm guard cartridge and heated to 32°C. Methanol was used as the mobile phase at a flow rate of 1 ml/min at 22 bar, and a UV detector measuring at 202 nm to determine the amount of cholesterol in each sample. The results were quantified against an external standard ranging from 0.05 to 2 mg/ml cholesterol and expressed as nanogram cholesterol per 1 × 10^6^ neutrophils.

Triglycerides, free fatty acids, monoacylglycerols, and phospholipids were analyzed by thin layer chromatography (TLC) based on a method described previously (35). Briefly, isolated lipid samples were loaded on silica gel plates (Merck, Germany) and separated based on polarity. Lipids were visualized by copper sulfate solution and the band intensities subsequently analyzed by CP Atlas (Lazer Software). Lipids were identified against a known standard. Each sample was analyzed in repetition.

### RNA Expression Analysis

RNA was extracted from 5 × 10^5^ neutrophils after incubation under normoxia or hypoxia for 2 or 3 h, with the RNeasy Micro Kit (Qiagen) as described in the user’s manual. RNA quality was tested with a bioanalyzer (RNA 6000 Pico Kit, Agilent) following the manufacturer’s instructions. Real-time PCR of reverse transcribed RNA (RT-qPCR) was designed to analyze expression of genes of interest and the housekeeping gene *rps9*. The respective primers are given in Table 1. The RT-qPCR was conducted as previously described (36) with the following modified program: initial denaturation at 95°C for 20 min and 40 cycles of denaturation at 95°C for 25 s, annealing at 58°C for 30 s, and amplification at 72°C for 20 s using an AriaMX Real-Time PCR system. Products were verified by melting curve analysis and 1.5% agarose gel electrophoresis. Data were normalized to a non-regulated housekeeping gene (*rps9*). The relative ΔCT values were determined for expression of the genes *hif-1*α, *ll-37*, and *vegf*. CT is the cycle number at the chosen amplification threshold, ΔCT = CT _gene (ll-37)_ − CT _reference (rsp9)_ and ΔΔCT = ΔCT _sample_ − ΔCT _calibrator_. The fold change in expression (2^−ΔΔCT^) was calculated as the read-out parameter. The calibrator was neutrophils under normoxia.

**Table 1:** **Oligonucleotide primers used in RT-qPCR**.

Gene	Primer sequence (5′-3′) forward	Primer sequence (5′-3′) reverse	Amplicon length (bp)
*rps9*	CTGACGCTTGATGAGAAGGAC	CTCATCCAGCACCCCAAT	87
*hif-1*α	GATGGAAGCACTAGACAAAGTTCA	ATCAGTGGTGGCAGTGGTAGTG	360
*ll-37*	GCCCAGGTCCTCAGCTACAAG	TGGTTGAGGGTCACTGTCCCC	260
*vegf*	ATGAACTTTCTGCTGTCTTGGGT	TGGCCTTGGTGAGGTTTGATCC	344

### Statistical Analysis

All experiments were performed at least three independent times unless indicated otherwise. Data were analyzed using Excel 2010 (Microsoft) and GraphPad Prism 6.0 (GraphPad Software). Differences between two or more groups were analyzed by using a two-way ANOVA with Sidak’s multiple comparisons test if not otherwise stated. The significance is indicated as follows: n.s., not significant, *p ≤ 0.05, **p ≤ 0.01, ***p ≤ 0.001, and ****p < 0.0001.

## Results and Discussion

### Low Oxygen Levels in *In Vitro* Neutrophil Suspension Culture

**Figure 1:**
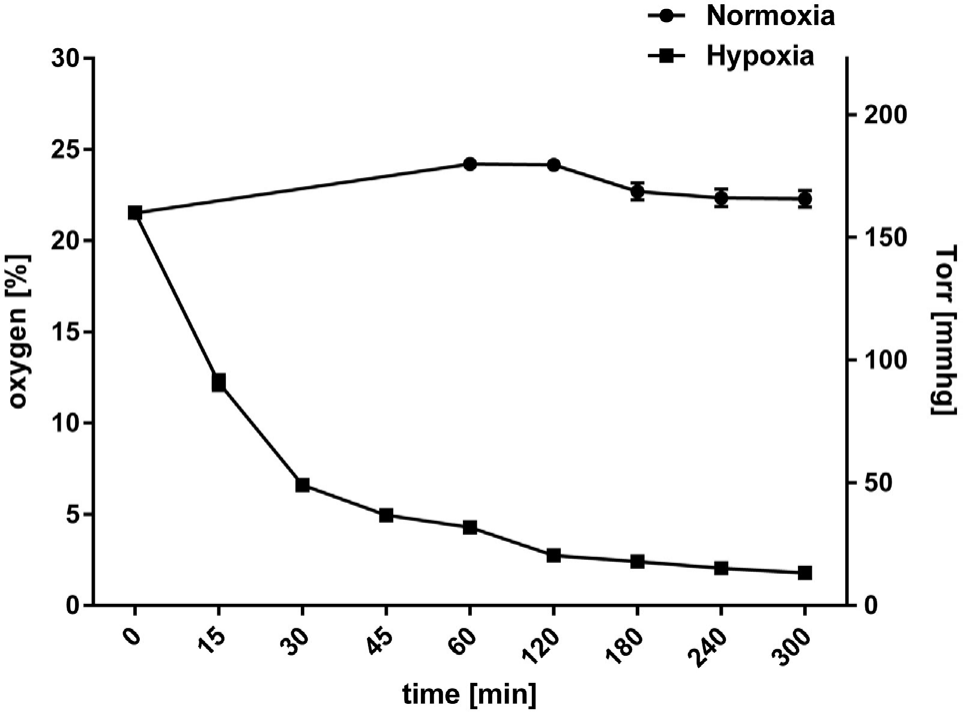
**Oxygen levels in neutrophil suspension cultures**. Neutrophils were incubated under normoxia (21%) or hypoxia (1%). Dissolved oxygen levels in neutrophil suspension culture were monitored for 5 h. Plotted values represent mean ± SEM and are displayed as % oxygen on the left *y*-axis and mmHg oxygen on the right *y*-axis. Starting at 21.5 ± 0.14% (normoxia) and 21.5 ± 0.21% (hypoxia) after 5 h, the oxygen level dropped to 1.79 ± 0.03% under hypoxia, while it stayed relatively constant around 22.3 ± 0.46% under normoxia.

At sites of infection and inflammation, the environmental oxygen concentration decreases and can drop to 1% due to invading pathogens and translocating immune cells, including neutrophils, with consequences for cellular functions (34, 37–39). To study the effect of hypoxia, freshly isolated human neutrophils were seeded and incubated under normoxic compared to 1% hypoxic conditions, and the oxygen level in the wells was measured over a time period of 5 h as described above. When cultured under normoxia, the neutrophil cultures maintained a constant oxygen level that reflected the atmospheric condition at around 165 mmHg (22.3 ± 0.46% O_2_). In contrast, hypoxic incubation decreased the dissolved oxygen level in the culture to less than 37 mmHg (4.9 ± 0.2% O_2_) within 45 min and resulted in an equilibration lower than 13.3 mmHg (1.79 ± 0.03% O_2_) within 5 h (Figure 1). Our results indicate that the applied experimental settings by incubating neutrophils under hypoxia decrease the oxygen level and reflect physiological oxygen conditions that may occur in infected tissue.

### Spontaneous and PMA-Induced Net Formation under Hypoxia Compared to Normoxia

To study the effect of hypoxia on the spontaneous and PMA-induced NET formation, neutrophils were preincubated for 2 h at the defined oxygen concentration (normoxia and hypoxia, as shown in Figure 1). After an additional 3 h of incubation in the presence or absence of PMA, the neutrophils were analyzed for their NET-releasing capability.

**Figure 2:**
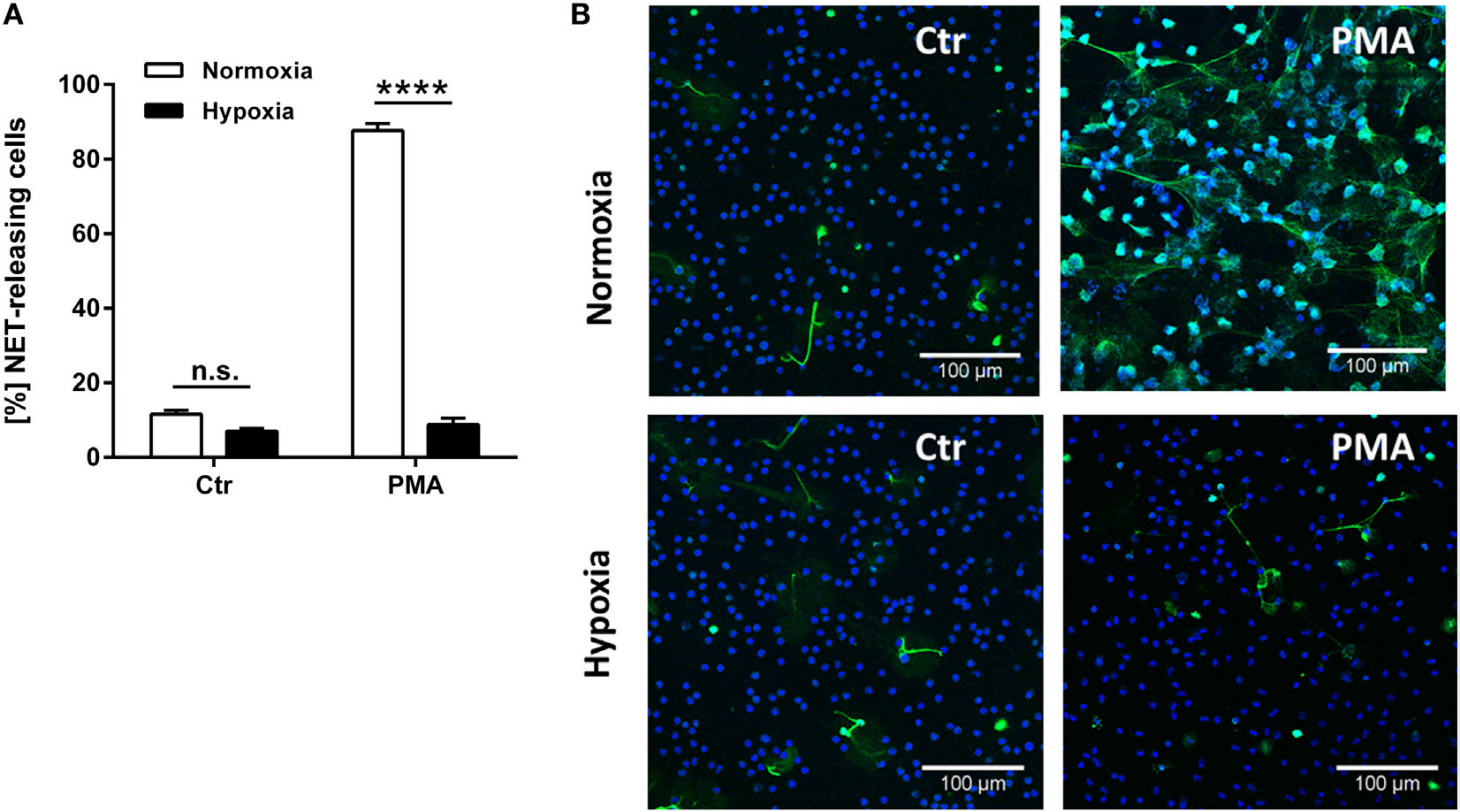
**Spontaneous and PMA-induced NET-release is decreased under hypoxia**. Neutrophils were incubated for 2 h under normoxia or hypoxia before treatment with 25 nM PMA for additional 3 h. Formation of NETs was visualized using a monoclonal antibody against histone–DNA complexes (green) in combination with DAPI to stain the nuclei (blue). **(A)** Under normoxia NET-release was stimulated with PMA in 87.7 ± 1.8% of the cells, while 11.6 ± 1.0% of the neutrophils formed NETs spontaneously. Incubation under hypoxia decreased the spontaneous NET-release to 7.1 ± 0.7% and abrogates the PMA-stimulatory effect to a rate of 8.8 ± 1.7%. **(B)** Representative fluorescence micrographs depicting NET-release of neutrophils. Statistical analysis of was performed with one-tailed paired Student’s *t*-test.

An amount of 11.6 ± 1.0% of untreated neutrophils spontaneously formed NETs under normoxia (Figure 2). Interestingly, incubation under hypoxia distinctly reduced the number of spontaneously NET forming neutrophils to 7.1 ± 0.7% (Figures 2A,B).

However, as previously published (1, 3) and confirmed here (Figure 2), approximately 90% of cells were found to release NETs after incubation at an atmospheric oxygen concentration in response to PMA. Importantly, hypoxic pretreatment of neutrophils completely abolished the PMA-induced NET formation seen at normoxic conditions (Figure 2). It is well known, that the PMA-dependent NET formation is based on the generation of ROS (1, 7). In good correlation to these findings, Kirchner et al. (40) recently confirmed that the formation of ROS is blocked at 2% O_2_ and also leads to an abrogated NET-release. Furthermore, a broad spectrum of antioxidative substances such as flavonoids, vitamin C, and aminosalicylic acid were also shown to inhibit NET formation through the reduction of ROS (41).

### Gene Expression of *hif-1α* and Target Genes

As already mentioned in the Section “Introduction,” HIF-1α is an essential regulator to modulate cellular stress responses to low oxygen conditions and also has been shown to modulate the formation of NETs (22, 25). Therefore, the mRNA expression of this transcription factor as well as two of its target genes (*vegf* and *ll-37*) (42–44) were investigated in neutrophils incubated under normoxia versus 1% hypoxia with RT-qPCR.

**Figure 3:**
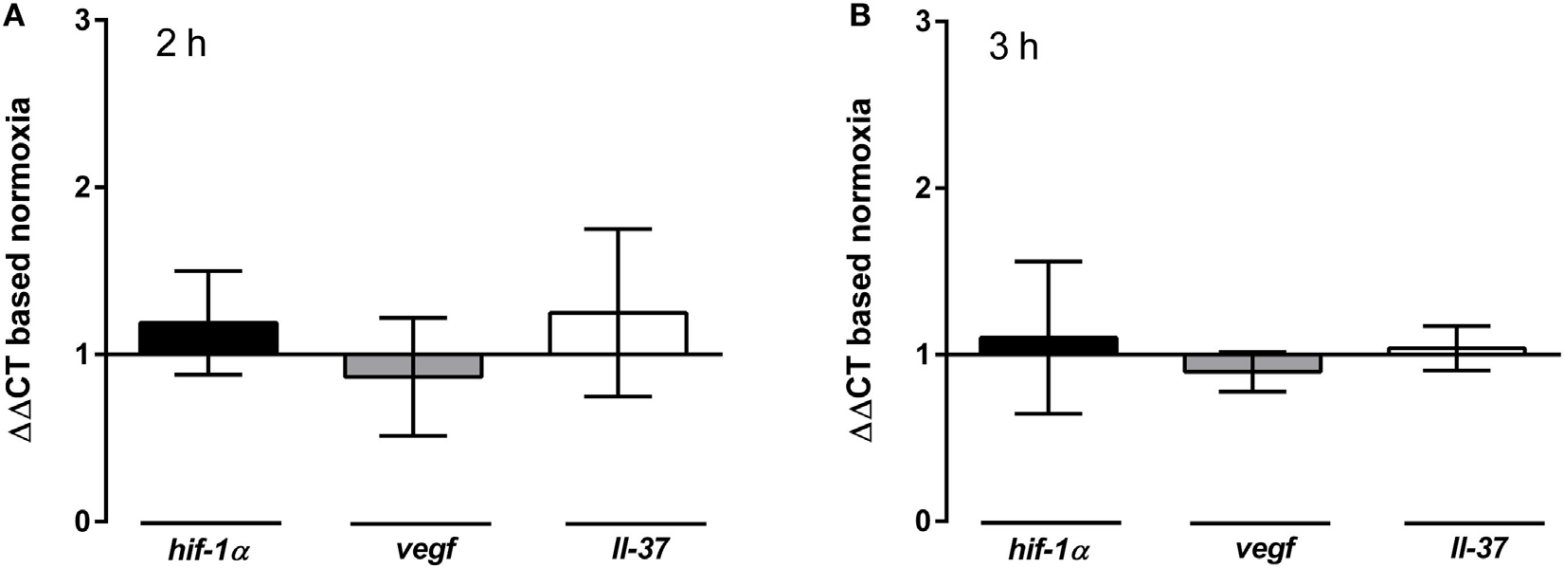
**Gene expression of *hif-1*α and target genes**. Neutrophils were incubated under normoxia or hypoxia for 2 h **(A)** and 3 h **(B)** before RNA was isolated and gene expression of *hif-1*α and its target genes *vegf* and *ll-37* was analyzed by RT-qPCR. Data were normalized to the non-regulated housekeeping gene *rps9*. The *x*-fold changes of the values from samples incubated under hypoxia were calculated against the normoxic samples. Independent of the time point, there was neither a regulation of *hif-1*α nor of its target genes *vegf* and *ll-37* detectable, as all ΔΔCTs remain below 2 [*hif-1*α: 1.19 ± 0.3 (2 h) and 0.1 ± 0.5 (3 h); *vegf*: 0.87 ± 0.4 (2 h) and 0.9 ± 0.1 (3 h); *ll-37*: 1.25 ± 0.5 (2 h) and 1.0 ± 0.1 (3 h)].

As depicted in Figure 3, the expression level of *hif-1*α or its target genes did not change neither after 2 h (Figure 3A) nor after 3 h (Figure 3B) incubation under hypoxia compared to normoxia. All ΔΔCT values remained around 1 meaning that there were no differences in the gene expression levels of the neutrophils that were cultivated under normal oxygen level in comparison to those cultivated under low oxygen levels. Therefore, short-time treatment at 1% hypoxia for 2 or 3 h might not be the optimal model to study the HIF-1α-dependent response of neutrophils. Moreover, HIF-1α might not be responsible for the altered NET-phenotype under hypoxia shown in Figures 2A,B. It seems that neutrophils need an additional trigger to induce the expression of *hif-1*α as hypoxia alone does not alter the expression level under the selected conditions (Figures 3A,B). In good correlation to our RT-qPCR results, Kirchner et al. (41, 40) also exhibited that hypoxia alone did not stabilize HIF-1α protein level. Interestingly, those authors demonstrated that HIF-1α is stabilized under hypoxia as wells as under normoxia by stimulating neutrophils with PMA (41).

### Lipid Alterations

**Figure 4:**
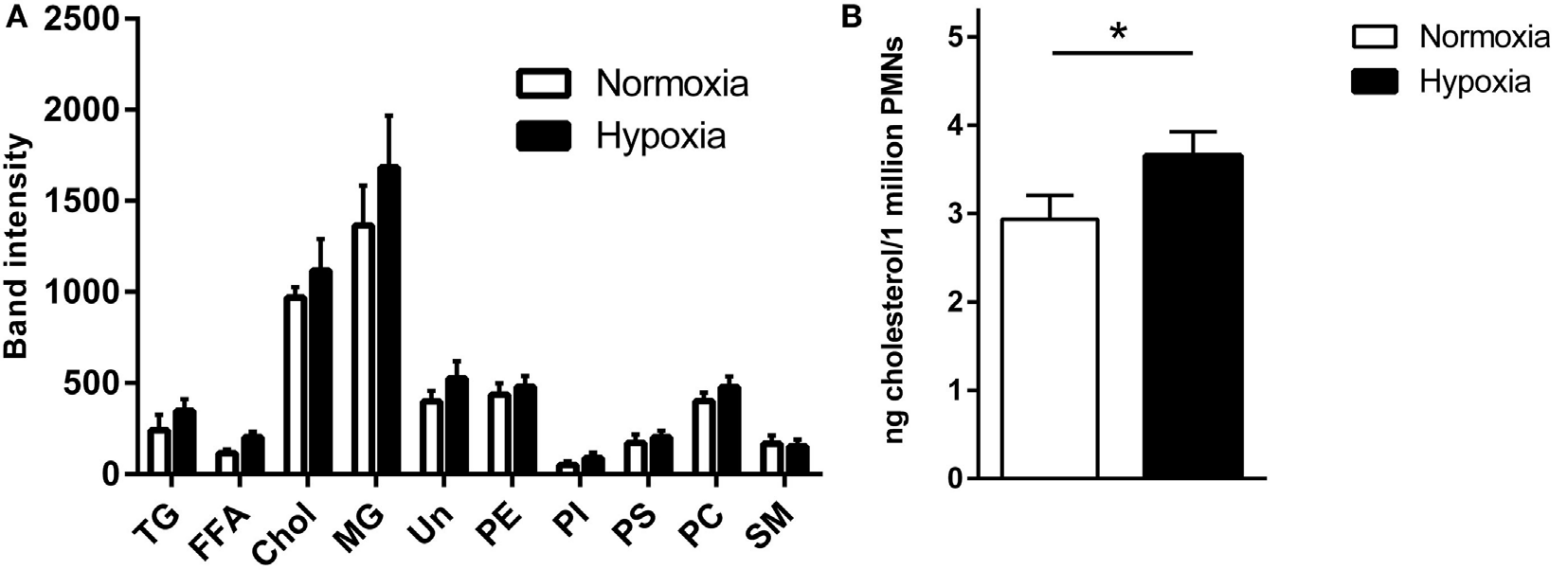
**The cellular cholesterol content increased under hypoxia**. Neutrophils were incubated in either 1 or 21% oxygen, and their lipid composition was analyzed by TLC **(A)**: triglycerides (TG), free fatty acids (FFA), cholesterol (chol), monoacylglycerols (MG), unknown (UN), phosphatidylethanolamine (PE), phosphatidylinositol (PI), phosphoserine (PS), phosphatidylcholine (PC), and sphingomyelin (SM). Furthermore, cholesterol content was analyzed *via* HPLC **(B)**. Results are expressed as nanogram cholesterol per 1 × 10^6^ neutrophils. A significant higher cellular cholesterol level was detectable after incubation under hypoxia compared to normoxia. Statistical analysis was performed with one-tailed paired Student’s *t*-test.

Previous data revealed that lipid alterations modulate the formation of NETs (45): decreased level of cholesterol mediated by methyl-β-cyclodextrin in primary blood-derived neutrophils led to increased spontaneous NET formation. Furthermore, pharmacological treatment of neutrophils with statins that block cholesterol synthesis also induce formation of NETs (46). Therefore, we here compared the lipid composition of the neutrophils when incubated under normoxia versus hypoxia by TLC. The data shown in Figure 4A demonstrate substantial lipid alterations comparing hypoxic and normoxic conditions, e.g., cholesterol. To verify the results, HPLC was used to quantify the cholesterol level in the cells. In good correlation to these data, a significant higher cholesterol level was found in cells after incubation under hypoxia compared to normoxia (Figure 4B) at the same time when decreased spontaneous NET formation was detectable in control cells (Figures 2A,B). Thus, it may be speculated that hypoxia-mediated changes in lipid content, e.g., cholesterol accumulation in the cell may contribute to altered spontaneous NET formation under hypoxia.

### *S. aureus*-Induced NET Formation

Even though the RT-qPCR results reveal no exclusive link of HIF-1α to hypoxia, our data are consistent: the absence of sufficient oxygen combined with the lack of HIF-1α signaling and increased cholesterol level hamper the formation of extracellular traps in neutrophils.

Nevertheless, we were interested if a bacterial stimulus could affect NET-release even under the above described condition. Therefore, it was of special interest to analyze the *S. aureus*-induced NET formation at low oxygen levels in more detail. The *S. aureus* wt strain and its nuclease-deficient Δ*nuc* mutant were used in parallel, since the Δ*nuc* mutant enables better visualization of full length NETs, which are not shortened by *S. aureus* nuclease. However, both strains are known to induce NET formation at similar level (30). Similar to the PMA-stimulated approach, neutrophils were incubated under hypoxia or normoxia for 2 h before bacteria were added, and the cells were further cocultivated with *S. aureus* for 3 h under consistent oxygen conditions.

**Figure 5:**
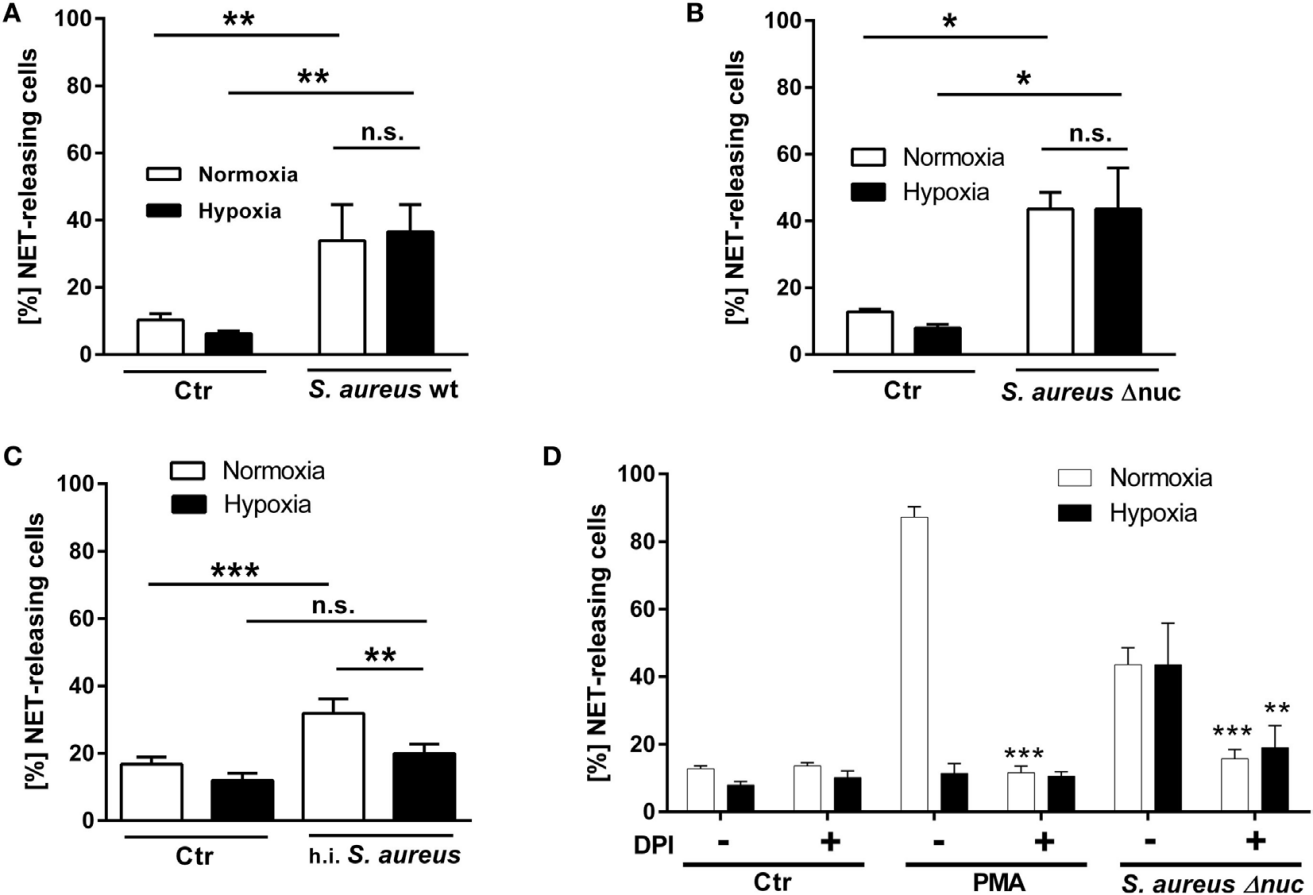
***S. aureus*-induced NET formation**. Neutrophils were preincubated under normoxic or hypoxic conditions for 2 h and further co-incubated for 3 h under the respective oxygen concentration with *S. aureus* wt (living and h.i.) and *S. aureus* Δnuc. Formation of NETs was microscopically analyzed and calculated as (%) NET-releasing cells. Neutrophils stimulated with *S. aureus* wt **(A)** and *S. aureus* Δnuc **(B)** release significantly more NETs compared to the amount of spontaneous NET formation both under normoxia as well as under hypoxia (normoxia: *S. aureus* wt: 33.9 ± 10.8% and ctr: 10.3 ± 1.9%/*S. aureus* Δnuc: 43.6 ± 5% and ctr: 12.8 ± 0.9%, hypoxia: *S. aureus* wt: 36.9 ± 12.8% and ctr: 8 ± 1.1%/*S. aureus* Δnuc: 43.6 ± 12.3% and ctr: 8 ± 1.1%). **(C)** The h.i. *S. aureus* caused significantly higher numbers of NET-releasing cells compared to untreated control under normoxia, while this difference was highly decreased under hypoxic conditions (normoxia: h.i. *S. aureus*: 31.8 ± 4.3% and ctr: 16.8 ± 2.1%, hypoxia: h.i. *S. aureus*: 20 ± 2.8% and ctr: 12.3 ± 2.1%), *n* = 2 independent experiments with each *n* = 6 images (total *n* = 12). **(D)** Neutrophils were treated with 10 μg/ml DPI to block NADPH oxidase activity. Formation of NETs was statistically analyzed compared to respective control in the absence of DPI by one-tailed unpaired Student’s *t*-test.

Interestingly, the amount of NET-releasing cells was not abrogated in the absence of O_2_: living bacteria induced comparable amounts of NET-releasing cells under normoxia as well as under hypoxia (Figure 5A: *S. aureus* wt: 33.9 ± 10.8% normoxia compared to 36.6 ± 8.0% hypoxia; Figure 5B: *S. aureus* Δ*nuc*: 43.6 ± 4.96% normoxia compared to 43.6 ± 12.3% hypoxia). These results confirm the existing literature and emphasize that living *S. aureus* have the capacity to stimulate NET formation independent of oxygen concentration (47, 48). In contrast to viable *S. aureus*, h.i. *S. aureus* was no longer able to induce NET formation under hypoxia with the same efficiency compared to normoxia, indicating a different oxygen-dependent mechanism triggered by dead *S. aureus* (Figure 5C: 31.8 ± 4.3% normoxia compared to 20 ± 2.8% hypoxia).

So far, different bacterial factors that are released by viable bacteria have been described to trigger the formation of NETs in response to *S. aureus* infections: the N-terminal ArgD peptides (49), leukotoxin GH (47), and Panton–Valentin leukocidin (PVL) (48). Importantly, those exotoxins trigger formation of NETs by completely different mechanisms. The PVL-mediated NET formation is described as a vesicular release of nuclear DNA by a mechanism independent of NADPH oxidase. In contrast, the leukotoxin GH and ArgD peptides-mediated NET formation is associated with cytolysis (no vesicular release of nuclear DNA), which is in accordance with the initial data from Fuchs et al. (3), who described the NADPH-oxidase-dependent formation of NETs in response to *S. aureus* as a novel cell death later called “NETosis” (50).

To further characterize the role of NADPH oxidase-mediated production of ROS in the *S. aureus*-mediated NET formation under 1% oxygen, we treated neutrophils with DPI to block NADPH oxidase. As shown in Figure 5D, treatment of neutrophils with DPI significantly decreased the *S. aureus* Δ*nuc*-mediated formation of NETs under hypoxia and normoxia as also previously shown by Fuchs et al. (3) under normoxia. These data indicate that under hypoxic as well as normoxic conditions, NADPH oxidase contribute to the formation of NETs in response to *S. aureus*. Since residual NET formation is still detectable in the presence of DPI (Figure 5D), additional pathways might also contribute to the phenotype as described by Pilsczek et al. (48). Under consideration of the lipid data shown in Figure 4, it might also be speculated that lipid alterations found under hypoxia lead to altered susceptibility of the neutrophil to bacterial exotoxins and/or oxidative stress as also shown by Chow et al. (46), and that these cellular changes modulate the neutrophil ability to release NETs. However, further studies are needed to prove this hypothesis.

Interestingly, our data obtained from the PMA- or *S. aureus*-stimulated neutrophils revealed a highly specific response, namely a characteristic NET formation level, to a distinct stimulus under various oxygen conditions. Whereas the NET formation mediated by viable *S. aureus* remains preserved under hypoxia (Figures 5A,B), the NET formation in response to PMA (Figures 1 and 5D) or dead *S. aureus* (Figure 5C) is reduced under hypoxia. However, the data shown here highlight that neutrophils can react completely different to the same trigger under hypoxia compared to normoxia. Since neutrophils are prominent immune cells found in inflamed tissue associated with low oxygen levels, hypoxia mimics the physiologic situation during an inflammation or infection much more accurately (34, 51). The described model should therefore be preferably used instead of the standard *in vitro* models, including the standardized NET induction assay, which are performed under normoxic conditions. Finally, in light of these results it should also be discussed if data obtained from studies performed under normoxia are really physiologically relevant. This is especially interesting since the formation of NETs was also implicated in the development of tumor-related diseases (52). Recently, Tohme et al. showed that NETs promote the development and progression of liver metastases after surgical stress. Importantly, in growing metastatic tumors, the authors found that intratumoral hypoxia accentuated NET formation (53). In line of this study, Alfaro et al. (54) demonstrated that tumor-produced IL-8 leads to extrusion of NETs in human myeloid-derived suppressor cells, which are considered an important T-cell immunosuppressive component in cancer-bearing hosts (54). Based on these data, it may be suggested that the elimination of NETs or pharmacological blocking of NET formation may reduce risks of tumor relapse. Thus, for a better understanding of the neutrophil biology as a target for new therapeutic interventions it is urgently needed to study its activity under specific physiological relevant oxygen conditions.

## Ethics Statement

This study was carried out in accordance with the recommendations of the Medizinische Hochschule Hannover Ethics Committee (Ethics Statement No. 3295-2016).

## Author Contributions

MK-B, LV, and HN: conceived and designed the experiments; LV, KB-H, DH, NB, SB, FR, and GB: performed the experiments; LV, MK-B, KB-H, NB, and GB: analyzed the data; HM, KB-H, and MK-B: wrote the paper.

## Conflict of Interest Statement

The authors declare that the research was conducted in the absence of any commercial or financial relationships that could be construed as a potential conflict of interest.
